# Human Pathogenic *Paecilomyces* from Food

**DOI:** 10.3390/microorganisms6030064

**Published:** 2018-07-05

**Authors:** Danielly C. Moreira, Manoel M. E. Oliveira, Cintia M. Borba

**Affiliations:** 1 Laboratory of Taxonomy, Biochemistry and Bioprospecting of Fungi, Oswaldo Cruz Institute, Oswaldo Cruz Foundation, Av. Brasil, 4365, 21045-900 Rio de Janeiro, RJ, Brazil; dcorrea@ioc.fiocruz.br; 2 Laboratory of Mycology, Evandro Chagas National Institute of Infectious Diseases, Oswaldo Cruz Foundation, Av. Brasil, 4365, 21045-900 Rio de Janeiro, RJ, Brazil; manoel.marques@ini.fiocruz.br

**Keywords:** *Byssochlamys*, food, beverage, spoilage products, hyalohyphomycosis

## Abstract

*Paecilomyces* spp. and *Byssochlamys* spp. are heat-resistant fungi important to industry because they can cause food and beverage spoilage, incurring economic loss. The consequences of food or beverage fungal colonization is the loss of nutritional value, structure and taste, and the possibility of producing toxic secondary metabolites that may result in medical problems. Furthermore, these fungi can infect animals and humans and it is unknown if contaminated foods may be fomites. *P. variotii* is the principal agent of food spoilage or contamination and it is most frequently associated with human hyalohyphomycosis with clinical manifestations including peritonitis, cutaneous and disseminated infections, among others. *Byssochlamys* spp. had not been identified as a cause of systemic infection until the case of a dog with a fungal infection, after immunosuppressive therapy. *P. variotii* has clinical importance because it causes severe infection in immunosuppressed patients and also because the number of immunocompetent infected patients is increasing. This review draws attention to the ability of these species to grow at high temperatures, to colonize food products, and to cause human disease.

## 1. Introduction

The genus *Paecilomyces* was originally described by Bainier [1] based on only one species, *Paecilomyces variotii*. Afterwards, the genus was studied by Hughes [2], Brown and Smith [3], Morris [4], Onions and Barron [5], de Hoog [6] and Samson [7]. Fifteen species were described on account of the morphology in pure culture [7]. Currently, the genus *Paecilomyces* has 145 epithets assigned in the Index Fungorum (www.indexfungorum.org); however, many of them are known to be taxonomic synonyms or questionable taxa [8].

Fungal species with hyaline to yellowish, septate, hyphae mostly smooth walled, with conidiogenous structures consisting of verticillate or irregularly branched conidiophores bearing divergent whorls of branches and phialides with cylindrical or inflated basal portion and a long distinct neck, producing one-celled hyaline conidia in basipetal chains are grouped inside the genus *Paecilomyces* [9].The species can be grouped into two sections: *Paecilomyces* including some mesophilic to thermophilic species with yellow-brown to brown colonies and *Isarioidea*, including some mesophilic species with white or pale-colored colonies [7]. Some species of *Paecilomyces* represent anamorphs of *Byssochlamys* Westiling, *Talaromyces* C. R. Benj, *Thermoascus* Miehe, *Cordyceps* Fr., and *Torrubiella* Boud [7,10].

Mycologists have traditionally used morphological characters to identify fungi to the species level but they can often be mislead due to hybridization, cryptic speciation, and convergent evolution [11]. Molecular tools (DNA sequence-based methods) have been useful to solving doubts in the identification process at the species level. Analysis of the 18S ribosomal DNA (rDNA) demonstrated that *Paecilomyces* can be considered polyphyletic across two *Ascomycota* orders, the *Eurotiales* and the *Hypocreales* [12]. The type of species of the genus is *Paecilomyces variotii*, which has a sexual *Byssochlamys* state.

Samson et al. [13] studied the genus *Byssochlamys* and its *Paecilomyces* anamorphs using a polyphasic approach by analyzing the internal transcribed spaces (ITS) region, partial sequencing of β-tubulin and calmodulin genes, macro- and micromorphology, and extrolite profiles (i.e., secondary metabolites). The results revealed that *Byssochlamys* includes nine species, five of which are teleomorphs (*B. fulva*, *B. laguncularieae*, *B. nivea*, *B. spectabilis*, *B. zollerniae*) while four are strictly anamorphic (*P. brunneolus*, *P. divaricatus*, *P. formosus*, *P. dactylethromorphus*). In addition, these authors divided *P. variotii* complex into four species, *P. divaricatus*, *P. formosus*, *P. dactylethromorphus* and *P. variotii*.

*Paecilomyces* species are often recovered from soil and air and can be found in acidic habitats and tolerating microaerophilic conditions. They may cause deterioration of grain, food, and paper and produce mycotoxins. Certain species have clinical importance because they produce infections in immunocompromised and immunocompetent patients [9].

The aim of this review is to add information about *Paecilomyces* from food and the possibility to cause medical problems to special issue Human Pathogenic Filamentous Fungi from Food [14]. 

## 2. *Paecilomyces* in Food

Fungi can grow on some food products. The result of food colonization can be the loss of nutritional value, structure and taste of the food, and the possibility of producing toxic secondary metabolites that may result in medical problems [15]. 

Concern has recently been raised over the possibility of fungi in food acting as pathogens in immunocompromised patients, but this issue has received little attention from food mycologists. According to Leong et al. [16] there are, at least, four cases of mycosis linked to fungi present in foods, including a fatal mycosis in a premature neonate. 

*Paecilomyces* spp. and *Byssochlamys* spp. are among the heat-resistant fungi important to the food and beverage industry because they can resist heat treatments used for food/beverage processing and can grow and spoil the products during storage at room temperature [17,18,19]. Dairy products, such as pasteurized milk, cream cheese, and heat-treated dairy beverages, are also spoiled by these species [20]. Furthermore, spices and herbs, including ground red pepper are easily contaminated by fungi, *Paecilomyces* among them, producing mycotoxins harmful to humans and animals [21]. Studies have provided evidence of the potential risk of long-term storage of maize seeds infected with *Paecilomyces* species [22,23], but the adverse human health effects are still unknown.

*P. variotii* is reported as the principal agent of food spoilage or contamination. Its presence in pasteurized beverages causes great economic losses [17]. It can also be found in margarine, processed cheeses, dried fruits, cereal/grains, rye, fruits, meet products, nuts, oils, and seeds [9,14,24]. This species is able to degrade the food preservatives sorbic acid, benzoic acid, and propionic acid, which results in changes in the smell of food products [15].

In a recent study, it was shown that *P. variotii* was the third most frequent thermophilic taxon isolated from spoiled Moroccan olive and olive cake [25]. Alkenz et al. [26] when isolating and identifying fungi from wheat flour, couscous, rice, and macaroni, found *P. variotii* in couscous and rice and *P. lilacinus* (=*Purpureocillium lilacinum*) in couscous.

*Byssochlamys* species have been studied as food spoilage agents for many years [27,28,29,30]. The first documented fruit spoilage outbreaks attributed to *Byssochlamys* occurred in England in the 1930s and at that time, it was thought that the organism was confined to that country [28]. However, *Byssochlamys* species have been isolated from food in different countries, France, Switzerland, Netherlands, USA, China, Belgium, Canada, among others [13].

*B. nivea* (anamorph *Paecilomyces niveus*), *B. spectabilis* (anamorph *Paecilomyces variotii*) and *B. fulva* (anamorph *Paecilomices fulvus*) have been frequently associated with fruit juice spoilage [15,29]. The ability to survive heat treatment is due to the production of ascospores. These species produce ascospores capable of surviving 100 min at 85 °C and their heat resistance generally increases with the increase of sugar concentration in the surrounding medium [30,31]. Additional factors are pH and organic acids, which counteract heat resistance of ascospores, but only at a pH lower than 4 [15].

According to Engel and Teuber [32], *B. nivea* is also detected in raw milk, fresh cheese, and fermented milk. Prolonged storage and inadequate cooling (>12 °C) of these products allow growth and development of *Byssochlamys* colonies.

In Brazil, heat resistant fungi have been isolated from different commercial juices such as, pineapple [33], apple [34,35], caja, umbu (typical Brazilian fruits) [36], passion fruit [37], and *Byssochamys* species are the most frequent. In the USA, the most common cause of beverage spoilage is *B. spectabilis* [38]. 

## 3. Pathogenic *Paecilomyces* spp.

Respiratory symptoms caused by fungi in grain dust during harvesting among farmers with *Paecilomyces* spp. as one of the isolated species have been recorded [39]. Therefore, these fungi can be classified not only as spoilage microorganisms, but also as potential sources of public health problems [40,41].

Some species within the *Paecilomyces* genus have clinical importance because they produce infections in immunocompromised and immunocompetent patients. *Paecilomyces variotii* is the species most frequently associated with human and animal diseases [9]. And, as described in section 2, it is a species also reported as an agent of food spoilage or contamination. Its thermotolerant nature is suggested to contribute to its pathogenic potential [42].

On the other hand, *Byssochlamys* spp. had not previously been identified as a cause of systemic infection in animals or humans until the case reported by Atencia et al. [43] of a dog with an unusual fungal infection.

Borba and Brito [9], revising the literature from 1959 to 2014, found 55 reported human cases of hyalohyphomycosis caused by *P. variotii* presenting different clinical manifestations associated with endocarditis, peritonitis, pyelonephritis, sinusitis, pneumonia, endophthalmitis, osteomyelitis, cutaneous, and disseminated infections, among others, where peritonitis was the most common. New cases were reported and peritonitis was the most common clinical manifestation followed by pneumonia (Table 1). 

A special case is *Purpureocillium lilacinum* [55], known until 2011 as *Paecilomyces lilacinus*. We are including this species here because even today many authors cite it as *Paecilomyces lilacinus* [56,57]. Similar to species belonging to the genus *Paecilomyces*, *Purp. lilacinum* is considered an airborne fungus, cosmopolitan, saprophytic, and frequently detected in environmental soil samples. It can cause deterioration of grains, food, and paper [58,59]. Although little attention has been given to “*Purp*. *lilacinum* and food” it should not be excluded. It has been found in samples of couscous [26], and recovered from contaminated skin creams, lotions used clinically, catheters and plastic implants [9]. *Purp. lilacinum* is able to infect (a) immunocompetent and immunocompromised humans and (b) experimental animals [60]. Most reports are of human hyalohyphomycoses [9]. 

Furthermore, *Purp. lilacinum* has been found in insect species in Pakistan [61] that could lead to food contamination from cross contamination from the infected insects. Additionally, edible insects are part of the subsistence diets of tribes in Africa and Australia and are very popular in Thailand. However, edible insects have not been sufficiently tested to determine the risk of disease transmission to humans [62].

Most clinical manifestations are associated with ocular and cutaneous/subcutaneous infections as described by Borba and Brito [9,63,64,65,66,67,68,69,70,71,72,73,74]. More recently, an onychomycosis [75] and a rare case of nasal perforation [76] were published.

There are publications about the use of *Purp. lilacinum* for the control of nematode pests and it is the most widely tested biological control agent for the management of plant parasitic nematodes [77,78]. *P. variotii* has also been evaluated on its ability for ovistatic and ovicidal effect on *Ascaris* eggs [79]. However, it is important to emphasize that there is a risk when working with these species. These isolates, used as biological control agents, could infect humans and animals causing opportunistic mycosis and the literature suggests that they represent a problem in patients with impaired immunological system. In contrast, the incidence of infections in immunocompetent hosts is increasing for both species [48,80].

**Figure 1:**
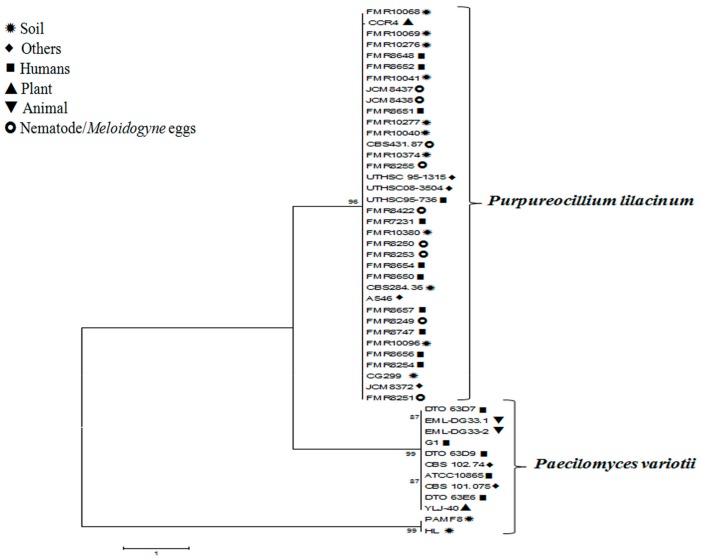
Phylogenetic tree of *Purpureocillium lilacinum* and *Paecilomyces variotii* isolates. Similarity between clinical and environmental *Purp. lilacinum* isolates is shown. *P. variotii* isolates show one possible differentiation between human and animal (clinical) isolates from soil isolates (environmental). The evolutionary history was inferred using the Neighbor–Joining method [81] among 48 taxa. The percentage of replicate trees in which the associated taxa clustered together in the bootstrap test (500 replicates) is shown next to the branches [82]. There were a total of 382 positions in the final dataset. Phylogenetic analyses were conducted in MEGA4 [83].

Molecular data presented by Luangsa-ard et al. [55] suggest that it is impossible to differentiate dangerous from beneficial isolates of *Purp. lilacinum*. We present in Figure 1 a comparison of nucleotide sequences of clinical isolates used in the papers referenced in this review with strains of *P. variotii* and *Purp. lilacinum* isolated from animals, plants, and soil (the sequences were obtained from GenBank). Figure 1 shows that isolates of *P. variotii* are distributed in two distinct clades with human and animal isolates in clade 1 and soil isolates in clade 2 and isolates of *Purp*. *lilacinum* show great similarity between clinical and environmental isolates. These phylogenetic trees demonstrate that it is difficult to separate *Purp. lilacinum* pathogenic from environmental isolates, which makes it a fungus that deserves greater attention.

## 4. Concluding Remarks

*Paecilomyces* spp. and *Byssochlamys* spp. represent some of the fungi that cause problems to the food and beverage industry and because these species are pathogenic to humans the contaminated food and beverage may become a probable source of infection. A better comprehension of the heat-resistance mechanisms of these species may provide insights for the development of new treatments to avoid food colonization and further investigations are necessary to understand the association of fungi with foods and the risks involved for humans.

## Figures and Tables

**Table 1:** Reported human cases of hyalohyphomycosis caused by *Paecilomyces variotii* between 2013 and 2017.

Infection	Number of Cases	Reference
Fungemia	1	Bellanger et al. [44]
Peritonitis	5	Uzunoglu and Sahin [45] Polat et al. [46] Torres et al. [47]
Pneumonia	4	Feldman et al. [48] Abolghasemi et al. [49] Kim and Williams [50] Steiner et al. [51]
Sepsis	1	Akhunov et al. [52]
Sinusitis	1	Swami et al. [53]
Subcutaneous infection	1	Vasudevan et al. [54]
